# L'Affaire Lazarus-Barlow

**Published:** 1914-01-17

**Authors:** 


### L'AFFAIRE LAZARUS-BARLOW.

THE public and the press may well be mystified by the extraordinary events of last week-end in connection with the treatment of cancer by radium. First of all, Dr. Lazarus-Barlow, for some years head of the Cancer Research Laboratories at the Middlesex Hospital, issued, or allowed to be issued, to the press a definite pronouncement that radium cures cancers which are inoperable surgically, and, therefore, almost beyond all hope of recovery. Dr. Lazarus-Barlow is a well-known scientific worker of eminence and experience; and for that reason anything he says carries weight. Had his views been communicated to the Royal Society of Medicine, or through a recognised medical journal, they would have been treated by his colleagues as at least worthy of discussion, and a helpful debate might have followed. As it is, the surgical staff of the Middlesex Hospital have felt obliged to sign a joint letter to the *Times* repudiating Dr. Lazarus-Barlow's optimistic views. In fact, the letter is more than a quip courteous: it almost amounts to the lie direct. Its natural result, one would imagine, must be the resignation by Dr. Lazarus-Barlow of his position.

If this quarrel affected no one but the professional gentlemen in question, there would be little need for comment here. But, unfortunately, it affects the public very nearly and intimately, as Dr. Lazarus-Barlow could surely have foreseen if he had stopped to think. Any quack who claims to cure cancer or consumption secures the public ear and makes a fortune nowadays. How much more, therefore, are the laity likely to entertain false hopes when an announcement such as this comes from the head of a Research Laboratory, well known for the sterling quality and quantity of work it has put forth on this very subject! That Dr. Lazarus-Barlow's views are premature and unduly optimistic is the verdict of practically every expert in radium therapy, as well as of those who specialise in cancer. The result of their publication in this unusual and improper way has merely been to raise hopes in the minds of thousands of relatives of patients dying of this dread malady—hopes which cannot be fulfilled and would have been much better left unawakened. That radium occasionally causes particular masses of cancer to disappear is undoubted; and it also very frequently relieves cancer pain, wholly or in part. But *cure* is another matter, any pronouncement upon which is unscientific and unjustifiable at present, and must remain so for some years at least.

The other topic broached by Dr. Lazarus-Barlow was the excessive cost of radium, and this also has evoked correspondence which is of the utmost interest to institutional managers. In effect he charges the manufacturers of radium with an artificial inflation of the price of this substance, contrary to the interests of suffering humanity. The manufacturers reply that the present exceedingly high price is due to the ordinary laws of supply and demand, superadded to the lengthy and costly processes of extracting radium from the ores and purifying it. The exact truth lies midway between these two opinions. There has been manipulation of the price of radium; but a community which allows its Government to levy a 200 per cent. tax on ether and chloroform can hardly complain of a "corner" in radium. On the other hand, the supply is very limited, the demand is rapidly growing; and this is the chief factor in the rise in value. It has also to be noted that there is a strong probability of the therapeutic rays of radium being successfully produced from *x*-ray tubes sooner or later; so that manufacturers who lay down expensive plant for preparing radium from pitchblende are running a considerable risk of awaking some day to find it useless. Dr. Lazarus-Barlow's indictment of the manufacturers does not, therefore, succeed by any means in full, though it is certainly true that some institutions have been forced to pay more for radium than they ought to have done.

